# In Vitro Performance and Chemical Stability of Lipid-Based Formulations Encapsulated in a Mesoporous Magnesium Carbonate Carrier

**DOI:** 10.3390/pharmaceutics12050426

**Published:** 2020-05-06

**Authors:** Caroline Alvebratt, Tahnee J. Dening, Michelle Åhlén, Ocean Cheung, Maria Strømme, Adolf Gogoll, Clive A. Prestidge, Christel A.S. Bergström

**Affiliations:** 1 Department of Pharmacy, Uppsala Biomedical Centre P.O. Box 580, Uppsala University, SE-751 23 Uppsala, Sweden; caroline.alvebratt@farmaci.uu.se; 2 University of South Australia, UniSA: Clinical and Health Sciences, Adelaide SA 5000, Australia; tahneedening@ku.edu (T.J.D.); clive.prestidge@unisa.edu.au (C.A.P.); 3 ARC Centre of Excellence in Convergent Bio-Nano Science and Technology, University of South Australia, Adelaide SA 5000, Australia; 4 Division of Nanotechnology and Functional Materials, Department of Engineering Sciences, Uppsala University, SE-75121 Uppsala, Sweden; michelle.ahlen@angstrom.uu.se (M.Å.); ocean.cheung@angstrom.uu.se (O.C.); Maria.Stromme@angstrom.uu.se (M.S.); 5 Department of Chemistry-Biomedical Centre P.O. Box 576, Uppsala University, SE-751 23 Uppsala, Sweden; adolf.gogoll@kemi.uu.se; 6 The Swedish Drug Delivery Center, Department of Pharmacy, Uppsala Biomedical Centre P.O. Box 580, Uppsala University, SE-751 23 Uppsala, Sweden

**Keywords:** mesoporous magnesium carbonate, lipid-based formulations, solidification, lipid release, lipolysis, ^1^H nuclear magnetic resonance

## Abstract

Lipid-based formulations can circumvent the low aqueous solubility of problematic drug compounds and increase their oral absorption. As these formulations are often physically unstable and costly to manufacture, solidification has been suggested as a way to minimize these issues. This study evaluated the physicochemical stability and in vitro performance of lipid-loaded mesoporous magnesium carbonate (MMC) particles with an average pore size of 20 nm. A medium chain lipid was loaded onto the MMC carrier via physical adsorption. A modified in vitro lipolysis setup was then used to study lipid release and digestion with ^1^H nuclear magnetic resonance spectroscopy. The lipid loading efficiency with different solidification techniques was also evaluated. The MMC, unlike more commonly used porous silicate carriers, dissolved during the lipolysis assay, providing a rapid release of encapsulated lipids into solution. The digestion of the dispersed lipid-loaded MMC therefore resembled that of a coarse dispersion of the lipid. The stability data demonstrated minor degradation of the lipid within the pores of the MMC particles, but storage for three months did not reveal extensive degradation. To conclude, lipids can be adsorbed onto MMC, creating a solid powder from which the lipid is readily released into the solution during in vitro digestion. The chemical stability of the formulation does however merit further attention.

## 1. Introduction

The cost of developing drug therapies is continually increasing, and capitalized costs are currently around 1.8 billion USD for a large pharmaceutical company to bring a new molecular entity to the market [1]. More complex treatment regimens and increased safety demands from regulatory authorities—for example, the inclusion of more subjects in clinical trials—partly explain the increased costs [2]. However, with up to 90% of drug candidates in the pipeline being classified as poorly soluble, low aqueous solubility poses a major challenge to effective pharmaceutical oral product development [3,4]. Various advanced drug delivery systems have been introduced to overcome solubility challenges, including amorphous solid dispersions and lipid-based formulations (LBFs) [4,5,6,7].

Although several LBFs are currently marketed (e.g., Lipirex, Norvir and Sandimmune Neoral), the commercial development of LBF products remains limited. Costly manufacturing and poor physical and chemical stability are some of the challenges associated with LBFs [8]. To ease handling and hence reduce production costs, solidification of LBFs has been investigated. The solidification methods vary in both execution and complexity, but commonly applied methods include physical adsorption, freeze-drying, spray-drying and hot melt extrusion. Typically, the LBF is adsorbed onto a solid carrier material with a large specific surface area, high porosity, or both [9,10]. Common carriers for adsorption of LBFs include silicon dioxide, magnesium aluminometasilicate, calcium silicate, and porous dibasic calcium phosphate [8,9,11]. 

One mesoporous magnesium aluminometasilicate product, Neusilin, has demonstrated significant potential for lipid encapsulation [9,12]. Besides enabling the creation of solid LBFs by simple physical mixing of LBF and the porous carrier [13,14,15], the solidification of LBFs using Neusilin also allows manufacture of tablets by direct compression [12,16]. However, incomplete release of the lipid excipients, and thereby also encapsulated drug cargo, into the solution is an issue with these formulations [9,13,14,15]. The limited desorption of the LBFs from Neusilin particles has been shown to translate to a reduced performance in vivo, i.e., resulting in lower oral drug absorption compared to liquid LBFs [15]. Properties of the LBF such as hydrophobicity also affect the release of the drug into solution, and larger fractions of lipid in the formulations increase retention of drug in the solid LBF. Reduced release of drugs as a function of long-term storage of the formulations is also a problem with solid LBFs [13,14]. Gumaste et al. investigated the release of probucol from Neusilin US2 loaded with various self-emulsifying drug delivery systems. On day one, there was complete drug release (>80%), but after 60 days of storage (40 °C/60%RH), less than 10% of the drug was released into the dissolution media by the worst performing formulation [13].

Due to these aforementioned limitations, new carriers to solidify LBFs are highly warranted. In 2016, mesoporous magnesium carbonate (MMC) was introduced as a viable drug carrier due to its ability to stabilize drug molecules in a non-crystalline form. Originally, MMC particles were synthesized with a pore size <6 nm, but particles with larger pore structures up to ~20 nm have since been developed [17,18]. The larger pore size resembles that of Neusilin US2 (~15 nm) [13,14], but with possible advantages over Neusilin. Accordingly, the overall aim of this study was to explore MMC as an excipient in solid lipid-based formulations. Our hypothesis was that the combination of the dissolution-enhancing properties of MMC and the increased solubilization capacity contribution of LBFs would increase the amount of drug released and ultimately provide higher bioavailability for poorly water-soluble drugs. An MMC-based solid LBF was produced via physical adsorption and was physicochemically characterized. Alternate solidification methods were also evaluated for the manufacturing of the solid LBF. To characterize the lipid release from this new mesoporous carrier, all solid LBFs were subjected to in vitro lipolysis studies to assess their dispersion–digestion properties.

## 2. Materials and Methods

### 2.1. Materials

MMC with a mean pore size of ~20 nm was synthesized in-house in accordance with previously published protocols [18]. Medium chain triglycerides (Captex 355) and medium chain mono- and diglycerides (Capmul MCM EP) were generously donated by Abitec (Janesville, VI, USA). According to the certificate of analysis provided by the manufacturer, the following composition of the glyceride chains of the triglyceride was found in the Captex: caprylic acid 60%, capric acid 40% and lauric acid <1%. Diethylene glycol monoethyl ether (Transcutol HP) was a kind gift from Gattefossé (Lyon, France). Fasted state simulated intestinal fluid (FaSSIF)-powder V1 was purchased from Biorelevant.com (Croydon, UK). Porcine pancreatin extract (activity equivalent to 8 × USP specification) was obtained from MP Biomedicals (Seven Hills, Australia). Ethanol and hydrochloric acid (36%) were acquired from Chem-Supply Pty Ltd. (Gillman, Australia). Polyethylene glycol (15)-hydroxystearate (Kolliphor EL), chloroform-d, dichloromethane, 4-bromophenylboronic acid, trizma maleate, calcium chloride dihydrate, and sodium hydroxide pellets were purchased from Sigma Aldrich (Castle Hill, Australia).

### 2.2. Preparation of Lipid-Loaded Mesoporous Magnesium Carbonate Particles

#### 2.2.1. Solidification of Lipid via Physical Adsorption

MMC was weighed into a vial and an equivalent quantity of Captex was slowly added via pipetting (1:1 *w/w* ratio). The sample was mixed with a spatula until a free-flowing powder was obtained [14,15].

#### 2.2.2. Solidification of Lipid via Freeze Drying

Captex was weighed into a vial and dispersed in ultrapure water at 10% *w/v* (i.e., 1 g Captex in 10 mL water) using a magnetic stirrer. MMC was added to the coarse emulsion to obtain a final Captex:MMC ratio of 1:1 *w/w*, and the resulting suspension was stirred for 15 min at room temperature. The suspension was then snap frozen using liquid nitrogen and placed in a freeze-dryer overnight (Lyph-Lock 6, Labconco, Kansas City, MO, USA). The freeze drying was performed under 0.01 mbar pressure and collector temperature was −45 °C.

#### 2.2.3. Solidification of Lipid via Solvent Immersion

Captex was dissolved in ethanol (60 mg/mL) via stirring for 15 min before an equivalent amount of MMC was added (final Captex:MMC ratio of 1:1 *w/w*). The suspension was stirred for an additional 3 h at room temperature. The ethanol was then evaporated under vacuum at room temperature overnight (Büchi Rotavapor RE, Büchi, Switzerland).

#### 2.2.4. Solidification Efficiency of Lipid-Based Formulation

To simplify ^1^H-NMR analysis of lipid digestion during in vitro lipolysis, this work focused mainly on MMC loaded with pure Captex. However, the efficiency of the MMC for adsorbing more complex lipid vehicles was also evaluated. A lipid-based formulation type IIIB-medium chain was selected as the model LBF [19]. The LBF was prepared by adding w/w Kolliphor EL (50%), Transcutol (25%), Captex (12.5%) and, Capmul MCM EP (12.5%) into a vial. The formulation was vortex-mixed and placed on a shaker overnight at room temperature. The LBF was then loaded onto the MMC using the three methods described in Section 2.2.1–Section 2.2.3. The following ratios of MMC:LBF was evaluated: 2:1, 1:1, 1:1.5, 1:2 *w/w*. The efficiency of the loading was evaluated based on visual inspection of the powder appearance and flowability.

### 2.3. Characterization of Captex-Loaded MMC

#### 2.3.1. Scanning Electron Microscopy

High-resolution scanning electron microscopy (SEM, Zeiss Merlin, Oberkochen, Germany) was used to study the surface morphology of the Captex-loaded MMC (CAP-MMC) and the LBF-loaded MMC (LBF-MMC). Double-sided adhesive tape was used to mount the samples, and the samples were then sputter-coated with a uniform gold layer of 5 nm depth. SEM-imaging was made at an accelerating voltage of 8kV [20].

#### 2.3.2. Thermal Gravimetric Analysis

Thermal gravimetric analysis (TGA) was carried out using a Mettler Toledo TGA/SDTA8511e balance (Columbus, OH, USA). The sample was added to an aluminum crucible and pretreated at 200 °C under an air flow of 40 mL/min for 10 min and then cooled to room temperature to remove adsorbed water. The water/solvent–free TGA curve of the sample was obtained by heating the sample from room temperature to 800 °C (10 °C/min) under an air flow of 40 mL/min. The Captex content in the CAP-MMC was estimated by comparing the weight loss on the TGA profiles of MMC and CAP-MMC (Equations (S1) and (S2)) [21].

#### 2.3.3. Nitrogen Sorption Analysis

A Micromeritics ASAP 2020 surface area analyzer (Micromeritics, Norcross, GA, USA) was used to record the nitrogen adsorption/desorption isotherm of the samples at −195.15 °C (*n* = 3). The specific surface area was determined using the Brunauer–Emmett–Teller (BET) equation. A Micromeritics SmartVacPrep 067 unit was used to pretreat the samples at 99.85 °C under dynamic vacuum (1 × 10^−4^ Pa) prior to the experiments [18]. The average pore size of a sample was determined using the density functional theory (DFT, N_2_ slit pore model was chosen according to Cheung et al. [18]) with the MicroActive software (Version 5.0, Micromeritics, Norcross, GA, USA). The total pore volume of the samples was calculated using the N_2_ equilibrium uptake at a relative pressure (p/p_0_) of 0.98.

#### 2.3.4. Fourier-Transform Infrared Spectroscopy

Fourier-transform infrared spectroscopy (FTIR) was performed with a Bruker ALPHA II coupled with a single reflection diamond attenuated total reflection module (Platinum ATR, Billerica, MA, USA). Prior to the measurements, 24 background scans were recorded and the ratio between the sample spectrum and background (reference) spectrum was calculated. For each spectrum (400–4000 cm^−1^), 24 scans were recorded with a resolution of 4 cm^−1^. Origin 2018 (OriginLab Corporation, MA, USA) was used to analyze the spectra.

### 2.4. Modified Lipolysis of Captex-Loaded MMC

The CAP-MMC was prepared using the three methods stated in Section 2.2.1–Section 2.2.3. A lipolysis buffer containing 50 mM Tris-maleate, 150 mM NaCl and 5 mM CaCl_2_ was prepared, and pH was adjusted to 6.5 [22]. The buffer was supplemented with FaSSIF-powder (3.0 mM sodium taurocholate and 0.75 mM lecithin) in accordance with the protocol from the supplier (Biorelevant.com). Pancreatic enzyme extracts were prepared by weighing porcine pancreatin into a vial and adding lipolysis buffer (0.2 g pancreatin per mL buffer). The extract was then placed on a magnetic stirrer for 15 min. Thereafter, the extract was centrifuged (1470× *g* at 4 °C for 20 min) and the supernatant decanted into a new tube [23].

Lipolysis was performed as described in previously published protocols [24,25]. In short, a Titrando 902 pH stat titration apparatus (Methrome, Switzerland) was used, and all experiments were conducted at 37 °C. For each experiment, the equivalent of 150 mg Captex was weighed into the lipolysis vessel and dispersed in 27 mL of preheated FaSSIF medium for 10 min, thereby forming a crude emulsion. Digestion was initiated by the addition of 3 mL pancreatic extract, resulting in a final lipase concentration of 1000 TBU per mL lipolysis volume. The pH was monitored and 0.6M NaOH was continuously titrated via an auto burette to maintain a constant pH of 6.5 ± 0.01. Samples of 1.5 mL were withdrawn at predetermined time points (0, 2, 5, 10, 30, 60 min), and added to Eppendorf tubes containing 15 µL 0.5M 4-bromophenyl boronic acid to inhibit further enzymatic digestion. The samples were then centrifuged at 35,170× *g* at 37 °C for 15 min. The supernatant was collected and stored in the refrigerator (~4 °C) prior to ^1^H-NMR analysis (see Section 2.5). Based on the amount of NaOH added during the lipolysis experiment, the extent of lipid hydrolysis was also calculated. The digestion was evaluated for the CAP-MMC manufactured by all three techniques (described in Section 2.2.1–Section 2.2.3). When CAP-MMC was studied, 300 mg of the formulation (equivalent to 150 mg Captex) was added to the preheated FaSSIF media and the enzyme extract was added immediately, i.e., no dispersion phase was implemented due to the dissolution of MMC. The lipolysis medium was titrated with 1M HCl to maintain a constant pH and to counteract the pH increase associated with the dissolution of the MMC [26].

### 2.5. ^1^H-NMR Sample Preparation and Spectra Acquisition of Lipolysis Samples.

Sample preparation was performed based on previously developed protocols [27,28]. In short, the lipolytic components within the supernatant of the centrifuged lipolysis samples were extracted using dichloromethane. Dichloromethane (2.25 mL) was added to each 1.5 mL lipolysis sample, which was then manually shaken for 2 min before being poured into a separation funnel. After settling for ~30 min, the organic phase was collected into a round-bottom flask and the dichloromethane was evaporated at room temperature (Rotavapor RE, Büchi, Switzerland). The resulting lipid film was dissolved in 800 µL of deuterated chloroform and transferred to an NMR tube. ^1^H-NMR spectra of the digested lipid were obtained using a Bruker Ascend™ 500. The following acquisition parameters were used: spectral width 10,000 Hz, relaxation delay 1 s, number of scans 64, acquisition time 3.277 s and pulse width 30°. A reference sample of Captex dissolved in deuterated chloroform was also prepared (Supplementary Materials, Table S2).

#### 2.5.1. Evaluation of Lipolysis Products and Extent of Lipid Digestion

The specific peaks in the ^1^H-NMR spectra were integrated and assigned to protons of the lipid compounds in the medium (Table S1). The relative number of moles of the different lipolytic species in the samples was calculated using mathematical models previously developed by Nieva-Echevarría et al. and Joyce et al. (Equations (S3)–(S7)) [27,28,29]. The molar percentage of the different lipolytic molecules was calculated from the number of moles of each component and the acyl groups supported on different glyceride structures, i.e., triglycerides (TG), diglycerides (DG), or monoglycerides (MG) (Equations (S8)–(S12)) [27,28,29].

### 2.6. Stability Evaluation of Captex-Loaded MMC

The chemical stability of CAP-MMC was evaluated during a three-month storage period, using Captex as a reference. Amorphous material (here relating to the MMC) is more prone to undergo solid-state changes in humid conditions [30], and to minimize cofounding effects due to adsorption of water on the MMC the study was performed under dry conditions [31]. For each sampling point, 300 mg of formulation was prepared as described in Section 2.2.1 in semi-open vials and placed in a stability chamber containing phosphorus pentoxide and silica gel (<5% RH/25 °C). Samples were collected immediately after preparation, and after 1, 2, and 3 months of storage. At each sampling point, 30 mg formulation (or 15 mg, for the reference Captex sample) was weighed into Eppendorf tubes (*n* = 3) and 3 mL preheated FaSSIF was added. The samples were vortexed for 1 min, and placed on a shaker at 37 °C for 1 h. The samples were then centrifuged for 15 min at 37 °C (21,000× *g*) and the supernatant (~3 mL) was collected. The lipids were extracted and samples prepared as described in Section 2.5 prior to the ^1^H-NMR analysis. ^1^H-NMR spectra of the lipid components were obtained using a Bruker Avance Neo 600 (Billerica, MA, United States), and the spectra were recorded using a cryoprobe. The relaxation rate constants (T1) for all signals of interest were determined before analysis of the stability samples, and the relaxation delay adjusted to 5 × T1. The following acquisition parameters were used: spectral width 11,905 Hz, relaxation delay 8 s, number of scans 32, acquisition time 2.7525 s, and pulse width 30°. The relative percentage of the lipolytic species obtained within the aqueous phase was calculated as described in Section 2.5.1. A one-month stability study was also conducted with CAP-MMC in which the MMC was degassed under vacuum at 120 °C for 6 h, in order to remove adsorbed water (details found in Supplementary Materials).

### 2.7. Stastistical Analyses

The mean and standard deviation were determined for all digestion measurements. All statistical analyses were performed in GraphPad Prism 7.03 (GraphPad Software Inc., San Diego, CA, USA). Changes during storage in the TG content in Captex and CAP-MMC were evaluated using linear regression analysis. An un-paired t-test tested if the slopes were significantly different between the Captex and the CAP-MMC. Two-way ANOVA tests combined with Sidak’s multiple comparisons test were used to evaluate differences in lipid components of the Captex and the CAP-MMC observed during the stability study. The limit for significance was set to *p* < 0.05.

## 3. Results and Discussion

### 3.1. Characterization of Captex-Loaded MMC

**Figure 1:**
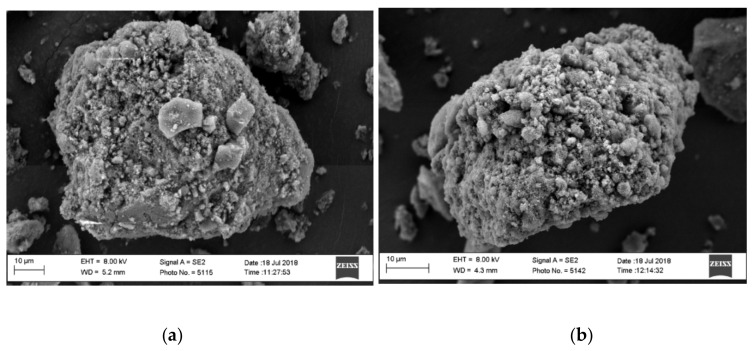
Scanning electron microscopy images of (**a**) mesoporous magnesium carbonate (MMC) and (**b**) Captex-loaded MMC (CAP-MMC) loaded via physical adsorption (1:1 *w/w* ratio). No morphological changes were observed on the surface of the MMC after loading with the lipid.

The physicochemical properties of the CAP-MMC manufactured by simple physical adsorption was extensively characterized herein to allow for comparison with solid LBFs produced similarly but using different mesoporous carriers [13,14,15,16]. After loading the MMC with Captex in a 1:1 *w/w* ratio, no visible impact was observed on the flowability of the powder. This is consistent with that reported for the mesoporous silicate, Neusilin US2, when loaded with LBFs in similar weight ratios via physical adsorption [12,13,16]. The SEM images demonstrated no morphological changes in the MMC particles after loading with Captex (Figure 1a,b). The lack of morphological changes suggests that the powder properties are unaffected by the adsorption of the lipid onto the MMC at this carrier:lipid ratio.

**Figure 2:**
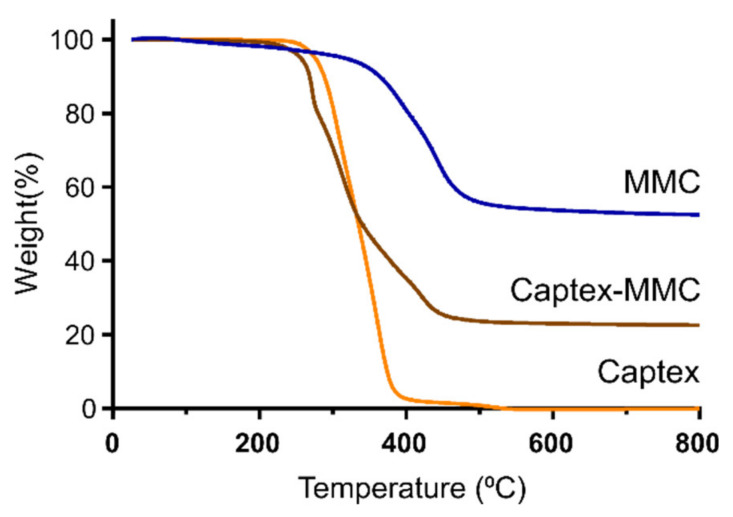
Evaluation of lipid load in CAP-MMC. Normalized mass thermal gravimetric analysis (TGA) curves of MMC, Captex, and CAP-MMC. The elevated weight loss seen for the CAP-MMC at 800 °C, compared to the MMC alone, can be attributed to the decomposition of the Captex.

The thermal gravimetric analysis (TGA) profiles demonstrated a significant difference in weight loss between MMC and the lipid-loaded CAP-MMC (Figure 2). As seen in Figure 2, the decomposition of the MMC to MgO took place over the temperature range, ~350–500 °C, in agreement with previous data [21]. The Captex lipid primarily decomposed between 200 and 400 °C. The TGA profile of CAP-MMC in Figure 2 is similar to those of MMC and Captex alone. Two mass decreases were observed, related to the decomposition of both Captex (at ~200 °C) and MMC (from around 350 °C). These were also reflected in the decomposition rates (dm/dt) (Figure S2), where the initial peak (at ~300 °C) correlated with the decomposition of Captex, and a shoulder on the peak at around 420 °C was consistent with that of the decomposition of MMC. The difference in weight observed at 800 °C for the MMC (52.5%) and the CAP-MMC (22.6%) can be attributed to the presence of the lipid (Figure 2). Based on this, the lipid content in the CAP-MMC was calculated to be 57.5% *w/w*. The calculated Captex weight percentage correlated well with the expected value of 50% *w/w* Captex:MMC. The MMC particles were heterogeneous in size, which can affect the lipid distribution within the bulk material. It has been seen that different particle sizes of the same lipid adsorbents with the same surface area exhibit different adsorption and release properties [9,32].

When the MMC was loaded with Captex, the surface area and pore volume of the MMC both decreased noticeably, which can be attributed to deposition of the lipid within the pores of the MMC (Table 1). Similarly, a significant reduction in pore volume (by approximately two thirds), was observed by Williams et al. when Neusilin US2 was loaded with an LBF in a 1:1 weight ratio [14]. It should be noted that different mesoporous carriers have different internal pore structures and size distribution; hence, some differences in the pore filling are to be expected. Neusilin US2 displays a similar pore volume (~1.2 cm³/g) as the 20-nm pore size MMC [14]; however, the specific surface area is slightly larger, ~300 m^2^/g (average value stated by supplier Fuji Chemical Industry).

The CAP-MMC sample still displayed some level of porosity after loading (Table 1). Apart from the detectable pore volume, which suggested that some pores on MMC were not filled, we noted no significant changes in the average pore size of MMC after loading with Captex (from ~26.4 to ~25.0 nm) (Table 1, Figure S1). This indicates that Captex loading occurred primarily by pore blocking and that the loading only occurred on some of the pores (i.e., not homogeneously)). As the remaining pores were effectively empty (and detected by N_2_ sorption), the detected pore size was therefore similar to the pore size of MMC. Note that if Captex was homogenously loaded in a layer on the surface of the pores on MMC (i.e., pore filling), the pore size of MMC-CAP would have decreased.

**Figure 3:**
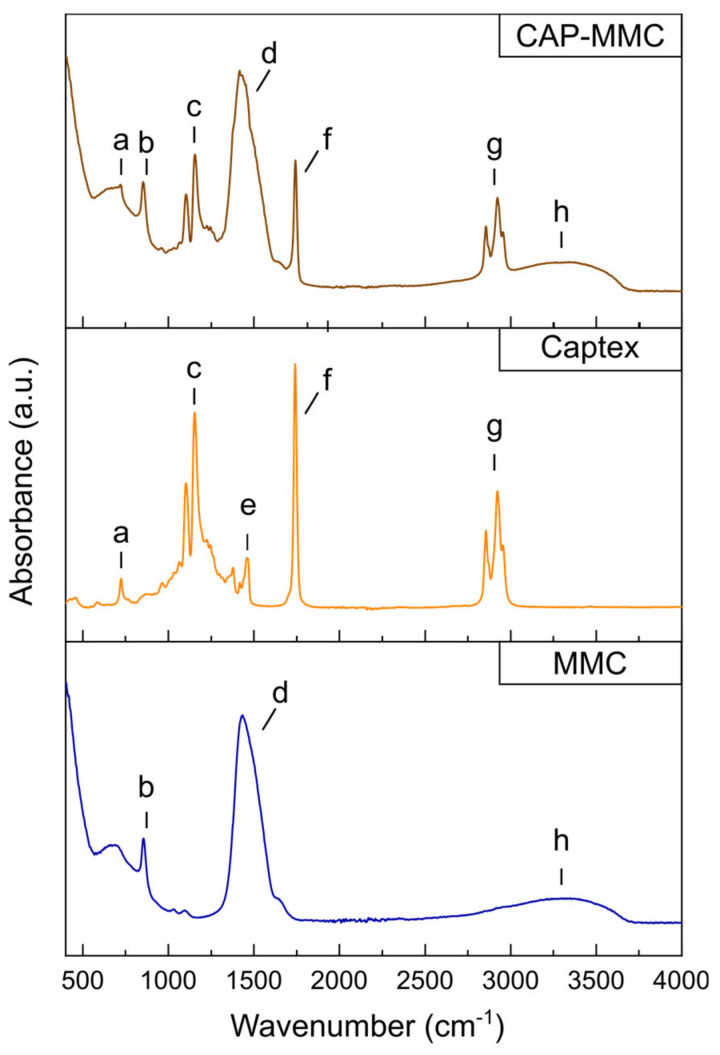
Evaluation of chemical interactions in CAP-MMC. Fourier-transform infrared spectroscopy (FTIR) spectra of Captex (orange), MMC (blue), and CAP-MMC (brown). Bands (**a**,**c**,**e**,**f**,**g**) can be attributed to molecular groups within the triglycerides in the Captex as follows: (**a**,**e**) CH_2_, (**c**) C-O, (**f**) C=O ester, and (**g**) CH_2_/CH_3_ [34]. Bands (**b**,**d**) are consistent with CO bonds within MMC. The broad band (**h**) at around 2700–3500 is consistent with hydrogen-bonded OH groups in MMC, which may be attributed to hydrogenated species of surface Mg(HCO3)_2_, Mg(OH)(HCO_3_) or adsorbed water, in the MMC [18,35]. No new chemical bonds were detected after the Captex was loaded onto the MMC.

FTIR spectra have previously been used to study interactions of lipids encapsulated in inorganic particle matrices (silicon dioxide nanoparticles, laponite platelets, and montmorillonite) [20]. In that study, a shoulder to the C=O ester stretch of the TG (at 1741 cm^−1^) was observed after encapsulation of the lipid in the smectite clay minerals, indicating a changed environment for the TG head groups. Both materials showed a higher retention of the TG in the particle matrix compared to the silica nanoparticles during 60 min digestion in fasted state simulated intestinal fluid, in which this alteration in the FTIR spectra was not observed. Like the silica nanoparticles, no alterations in the ester stretch at 1740 cm^−1^ were observed in the FTIR spectra of CAP-MMC (Figure 3, Table S3). The bands in the FTIR spectrum of CAP-MMC were consistent with the spectra of the raw materials, i.e., Captex and MMC (Figure 3). No new IR absorbance bands or band broadening were observed after adsorption of the Captex onto the MMC, indicating that no strong interaction e.g., chemical bonds were formed between the two components in the CAP-MMC (Figure 3). Furthermore, formation of hydrogen bonds is unlikely as no shifts in the bands assigned to relevant functional groups within the Captex and MMMC were observed (Table S3). However, the broad band attributed to hydrogenated species observed in the MMC, and thereby also the CAP-MMC, makes it impossible to completely exclude hydrogen bonding. Other interactions, such as ion–dipole and van der Waals attraction, may however have occurred between the lipid and the MMC. The absence of covalent bonds between Captex and MMC would suggest that the release of Captex is diffusion-controlled. This would be consistent with previous reports of drug-loading onto MMC [33]. The dissolution rate of the MMC will however strongly influence the release rate of the lipid. There were no changes in the FTIR spectra of the Captex and the CAP-MMC during three months of storage (Figure S2).

### 3.2. In Vitro Digestion of Captex-Loaded MMC

**Figure 4:**
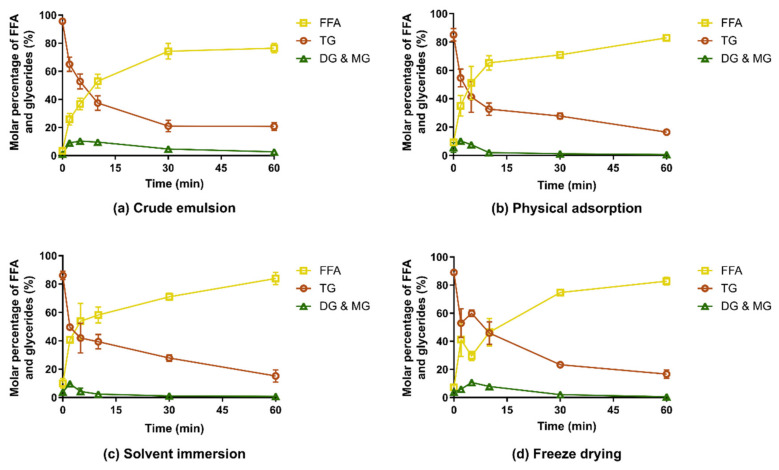
Relative molar percentage of the different lipolytic compounds during in vitro lipolysis of Captex and CAP-MMC, as determined using ^1^H-NMR spectroscopy (*n* ≥ 3). Digestion of (**a**) a crude emulsion of Captex formed upon dispersion of Captex in the lipolysis medium, and release and digestion of CAP-MMC prepared using: (**b**) physical adsorption, (**c**) solvent immersion, and (**d**) freeze drying. The digestion profiles of all three versions of CAP-MMC displayed a similar digestion pattern as the crude emulsion of the Captex. FFA: Free fatty acids, TG: Triglycerides, DG&MG: Di- and monoglycerides.

The digestion of the CAP-MMC differed from that of lipid-loaded porous silica containing a medium chain lipid with similar caprylic/capric fatty acid substitution of the TGs (Miglyol 812) [20,36]. In those studies, a significantly faster digestion of the lipid was reported for the lipid-loaded silica compared to a crude emulsion of the lipid. This can be attributed to an increased surface area of the lipids when adsorbed to silica and enhanced lipid-lipase interaction due to the hydrophilic nature of silicon dioxide [20]. In contrast, the release and digestion of lipid from CAP-MMC in our experimental setup was similar to the crude emulsion formed upon dispersion of the Captex in the lipolysis media (Figure 4). The digestion profiles were consistent for all CAP-MMC, regardless of manufacturing technique. This can be explained by the rapid dissolution of the MMC in the experimental setup; indeed, no MMC particles were seen within 10–15 min after addition of the CAP-MMC to the lipolysis vessel. The undigested TG content after 60 min was 15%–20% for all formulations. The formation of the free fatty acids (FFAs) was also consistent with this number, with final fractions of 75%–80%. However, lipid hydrolysis appeared to be slightly faster for CAP-MMC than in the crude emulsion. Initially, the Captex was more finely dispersed when loaded onto the MMC than in the lipid alone, but as soon as the MMC dissolved, this effect may be lost due to the coalescence of lipid droplets in the medium. The droplet size should therefore be similar for the two systems, as none of the formulations contained excipients that would result in emulsification and hence facilitate the formation of smaller, stabilized lipid droplets.

Issues related to the desorption of lipid components in porous carriers may be circumvented with the use of a soluble carrier. The dissolution of MMC appeared to result in a complete release of the lipids. This is in contrast to the incomplete lipid desorption seen for other LBF-loaded insoluble mesoporous carriers, e.g., Neusilin [13,14,15]. Dissolution of the MMC was rapid under the conditions of our modified lipolysis assay, in which the pH was maintained at 6.5 by titration of acid. However, the extent and rate at which this dissolution of MMC would occur in vivo has yet to be evaluated. The titration with HCl maintained lipolytic enzyme activity, but the buffering capacity in the regions of the gastrointestinal tract differs significantly from each other [37]. Since the activity of the pancreatic lipase is strongly influenced by the surrounding pH [38,39], changes in the microclimate around the particles may affect phenomena such as drug solubilization and digestion of the LBF, due to altered enzymatic activity.

For one of the formulations, freeze-dried CAP-MMC, an increase in triglyceride content—and conversely a decrease in the molar percentage of free fatty acids (FFAs)—was observed after 5 min (Figure 4d). This may be an artefact of the method, as ^1^H-NMR spectra only show the relative molar quantities. Nonetheless, this trend was observed for all replicates. One potential explanation is the size reduction of the MMC particles during manufacturing of the freeze-dried material. Fine particles would dissolve very rapidly, giving a burst effect of small lipid droplets rather than a continuous release of TG. Initially, digestion would then be very rapid due to an excess of enzyme. After the initial phase, the release of the TG would be controlled by the dissolution of the larger MMC particles, similar to the other two CAP-MMC systems.

The amounts of DG and MG were low for both the crude emulsion of Captex and all CAP-MMC formulations. This indicates complete hydrolysis of the TG molecules into one glycerol and three fatty acids, rather than formation of one MG and two fatty acids. Complete hydrolysis of the TG has been shown to occur to a larger extent in vitro compared to in vivo. It is however not possible to determine if complete hydrolysis has occurred by simple evaluation of the ^1^H-NMR spectra obtained in this study, since the highly polar glycerol molecules cannot be extracted from the samples using dichloromethane [28].

Our results are in contrast to Denning et al.’s results from ^1^H-NMR spectroscopy studies of lipolysis samples [20]. In their study, digestion of a coarse emulsion of a similar medium-chain lipid (Miglyol 812) showed significant amounts of both mono- and diglycerides after 60 min. However, the FFA fraction in the crude emulsion at 60 min observed herein using ^1^H-NMR (76.5% ± 3.4%) is consistent with our calculations based on the titration of NaOH during the lipolysis (77.1% ± 2.1% lipid hydrolysis), assuming complete hydrolysis of the TG, i.e., one TG forms three FFA and one glycerol molecule. Nevertheless, the absence of the DG/MG signals for the lipolysis samples seen in Figure 4 may however also be due to parameter settings for the ^1^H-NMR measurements. The parameters used for analysis of the lipolysis samples were based on a previously developed in-house protocol for the evaluation of the in vitro lipolysis samples (see Section 2.5), and the ^1^H-NMR parameters were not selected for our specific samples.

For all CAP-MMCs, the triglyceride content (85.1% ± 4.4%–87.7% ± 2.7%) at 0 min (i.e., before addition of pancreatic enzyme) was lower than for the Captex alone (95.6% ± 0.9%) indicating that the CAP-MMC was chemically unstable.

### 3.3. Chemical Stability of Captex-Loaded MMC

**Figure 5:**
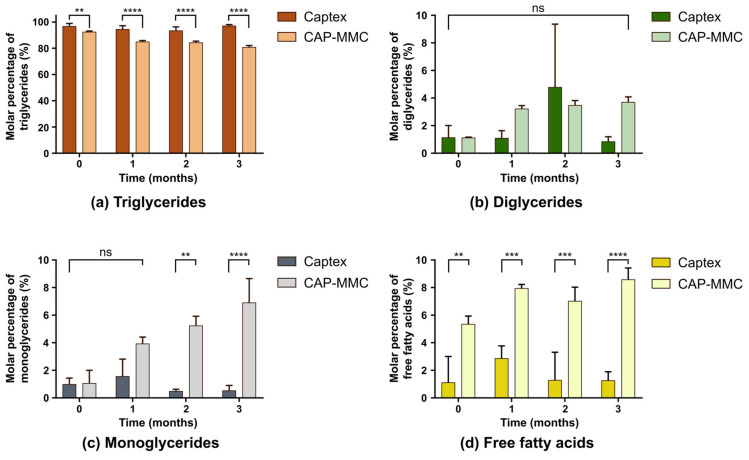
Molar percentage of each component in storage stability samples of Captex and CAP-MMC, showing each lipolytic component separately. The molar percentage of (**a**) triglycerides (TG), (**b**) diglycerides, (**c**), monoglycerides (MG) and (**d**) free fatty acids (FFA) was determined using ^1^H-NMR spectroscopy. Significant differences were seen in TG, MG and FFA content for the two formulations. (** *p* < 0.01 *** *p* < 0.001 **** *p* < 0.0001, ns: no significance)

Significant differences in the TG contents of Captex and CAP-MMC were detected even before the long-term storage commenced, at 0 months (Figure 5a).

Regression analysis of the TG change over time in the CAP-MMC showed a significant decrease in the TG content, which was not seen in the Captex alone. The decrease was more pronounced immediately after loading (92.5% ± 0.6% compared to 96.8% ± 2.2% in pure Captex) and during the first month (TG = 84.9% ± 1.0%). Only minor differences in the TG content in the CAP-MMC were observed after 2- and 3-months’ storage (84.3% ± 1.2% and 80.8% ± 1.3%, respectively). The FFA and MG also differed for Captex and the CAP-MMC, with higher amounts in the CAP-MMC (Figure 5c,d). For the CAP-MMC, MG and FFA contents appeared to increase over time, but these changes were not statistically significant. A lower TG content and correspondingly higher FFA content in the CAP-MMC suggested either that the Captex degraded within the pores of the MMC, or immediately after the CAP-MMC was dispersed in the biorelevant media. The latter is less likely, since a change in the TG content in the CAP-MMC was observed during storage.

There was no significant difference detected between the two formulations with regards to the DG content (Fig. 5b). The DG data obtained after two months of storage of Captex did however contain one experimental value that was relatively high as compared to the other two replicates (5b). As the TG amounts determined with this method depend on the relative amount of DG in the sample (Equation (S5)), this experimental value may, to some extent, affect the fraction of TG determined for 2 months of storage). The pH was not adjusted after the CAP-MMC was dispersed in FaSSIF in the stability study, and a small fraction of undissolved MMC was present in the pellet after the centrifugation. Lipid retention and preferential reabsorption of specific lipolytic components is seen with other solid carriers [20], which may also have been the case for MMC. The pellets formed after centrifugation were, however, similar for all of the CAP-MMC samples, and therefore do not explain the changes in the lipolytic composition found over time.

MMC has previously been reported to be incompatible with fenofibrate, a pro-drug containing an ester functional group [26]. Ester bonds are also present in TG, connecting the fatty acids to the glycerol backbone of the TG molecule. In alkaline environments, these ester bonds are susceptible to hydrolysis (saponification) and the addition of small amounts of water to dry alkaline lipid-mixtures increases the conversion of the esters [40,41]. The MMC was not preheated before the addition of Captex, so a small amount of water may have been adsorbed onto the surface. Adsorbed water could have catalyzed the hydrolysis, leading to the degradation of the TGs that were in direct contact with the MMC surface. According to the certificate of analysis, the Captex also contains trace amounts of water (0.01%). As the contact area between the TG and the MMC would not have changed during storage, this also explains why the TG content decreased between 0 and 1 months, and why there were no major differences in the 1–3 months’ CAP-MMC samples. However, preliminary results show that similar degradation is observed in CAP-MMC in which MMC was degassed before loading (Table S4), hence it is likely that the decomposition of CAP inside the carrier is not only a result of a chemical reaction driven by adsorbed water.

Surface modification may be a viable option to increase TG-stability. Various types of surface functionalization to alter the surface properties of mesoporous carriers, e.g., silica, have been extensively investigated [42,43], and surface modifications have also been evaluated for MMC. Recently, Vall et al. successfully functionalized MMC with aminosilanes and then loaded the modified MMC with salicylic acid [44]. By performing the amino modification, the risk of chemical reactions between the acid functional groups in salicylic acid and the carbonate groups in the MMC was reduced.

### 3.4. Solidification Efficiency of Lipid-Based Formulations

LBFs rarely contain a single lipid and the loading efficiency of MMC with a more complex LBF was therefore studied as a first study to understand the broader applicability of the MMC for manufacturing of solid materials containing LBFs. Based on the reduction in pore volume in the 1:1 *w/w* ratio MMC:Captex (Table 1), it seemed unlikely that much higher lipid-loads could be achieved. Indeed, the highest loading ratio achieved herein was 1:1.5–2 *w/w* MMC:LBF, depending on the solidification method used (Table S5). Higher LBF ratios resulted in poor flowability and/or lumps of LBF in the powder mixture. No morphological changes were observed in the SEM images of the LBF-loaded MMC, not even at the highest LBF-load achieved (Figure S4). The maximum loading ratio for MMC was in line with those obtained by Williams et al., who reported a complete pore filling for a 1:2 *w/w* ratio Neusilin:LBF [14]. When we used physical adsorption and solvent immersion, the maximum loading ratio was 1:1.5 *w/w* MMC:LBF.

A slightly higher loading was achieved for the LBF-MMC prepared using freeze drying (1:2 *w/w* MMC:LBF)(Figure S4). The higher degree of loading may have been due to the size reduction of MMC particles during the lipid loading process prior to the freeze drying (e.g., magnetic grinding and potential dissolution of the MMC in water). Even though the results indicate that the loading techniques used for preparation of the LBF-MMC only have a minor impact on the maximum loading degree of the MMC, further studies are needed to confirm this observation. Apart from the loading efficiency, the choice of loading method may also affect, e.g., distribution of the LBF within the carrier. Other important factors such as the characteristics of the LBF droplets released from the carrier are not covered in this study, but will significantly impact the suitability of MMC as a carrier for LBFs. Based on this initial work, MMC can be viewed as potential carrier for adsorption of LBFs. However, extensive work is needed to fully understand the usefulness of these formulations.

## 4. Conclusions

In this work, we developed and extensively characterized 20-nm pore-sized MMC loaded with a single lipid (Captex) or an LBF. Various loading methods were successfully applied to create solid LBFs, but the loading method only had a minor impact on the loading efficiency of the MMC. A modified lipolysis method, using titration with HCl and sample analysis by ^1^H-NMR spectroscopy, was developed to evaluate the digestion of Captex-loaded MMC particles. The release and digestion of lipid from CAP-MMC was similar to that of a crude emulsion of the lipid, due to dissolution of the MMC. Dissolution of the MMC also resulted in the complete release of the Captex into the solution, making it an attractive option for the oral delivery of lipids. However, free fatty acids were observed in the CAP-MMC already before digestion was initiated, indicating some degradation of Captex within the MMC pores. The extent of degradation was further evaluated during a three-month stability study, which revealed an initial degradation that slowed down following one month of storage. Despite the considerable benefits of a soluble carrier, the chemical instability of the CAP-MMC needs to be overcome if the MMC is to be used for solidification of lipids.

## Figures and Tables

**Table 1:** Brunauer–Emmett–Teller (BET) surface area, total pore volume (calculated at a relative pressure of 0.98), and peak density functional theory (DFT) pore size of MMC and CAP-MMC obtained using nitrogen sorption. Both the BET surface area and total pore volume were significantly reduced after loading with the lipid.

Sample	Specific Surface Area (m²/g)	Total Pore Volume (cm³/g)	Average Pore Size (nm)
MMC	196 (±0.20)	1.29 (±0.012)	26.4
CAP-MMC	42 (±0.64)	0.26 (±0.004)	25.0

## Supplementary Materials

The following are available online at https://www.mdpi.com/1999-4923/12/5/426/s1, Equations (S1) and (S2): Calculation of Captex content in CAP-MMC based on TGA curves, Equations (S3)–(S12): Calculations of the molar percentage of the different lipolytic molecules in the ^1^H-NMR samples, modified from previous mathematical models developed by Nieva-Echevarría et al. (2014, 2015) and Joyce et al. (2016), Table S1: Assignments of chemical shift of the ^1^H-NMR signal of the main protons of glycerides and free fatty acids during the in vitro digestion of Captex and CAP-MMC. Table S2: Molar percentage of the different lipid components in reference sample of Captex. Table S3: Band position and assignment of ATR FTIR spectra of Captex, MMC, and CAP-MMC. Table S4: Molar percentage of the different lipid components degassed CAP-MMC. Table S5: Loading efficiency of MMC using different loading techniques. Figure S1: Pore size distribution and cumulative pore volume of MMC and CAP-MMC determined using nitrogen sorption. Figure S2: Normalized mass thermal gravimetric analysis curves of MMC, Captex, and CAP-MMC. Figure S3: FTIR of Captex and CAP-MMC during three months’ storage in dry conditions. Figure S4: SEM image of LBF-loaded MMC (1:2 *w/w* ratio MMC:LBF) manufactured via freeze-drying.
