# Supplementary material for: In Vitro Performance and Chemical Stability of Lipid-Based Formulations Encapsulated in a Mesoporous Magnesium Carbonate Carrier
Source: Pharmaceutics. 2020 May 6;12(5):426. doi: 10.3390/pharmaceutics12050426 (PMC7284621; doi:10.3390/pharmaceutics12050426)
Supplement: Supplementary file 1 [file pharmaceutics-12-00426-s001.pdf]

pharmaceutics

Supplementary Materials: In vitro Performance and Chemical Stability of Lipid-Based Formulations Encapsulated in a Mesoporous Magnesium Carbonate Carrier

Caroline Alvebratt, Tahnee J. Dening, Michelle Åhlén, Ocean Cheung, Maria Strømme, Adolf Gogoll, Clive A. Prestidge and Christel A.S. Bergström

<u>Table of Contents:</u>

Calculation of lipid content in CAP-MMC (Equation S1-S2)

Assignments of chemical shift of the ^{1}H NMR (Table S1)

Calculations of the molar percentage of the different lipolytic molecules in the 1H NMR samples (Equation S3-S12)

Reference samples of Captex (Table S2)

Nitrogen sorption analysis of MMC and CAP-MMC (Figure S1)

Normalized mass thermal gravimetric analysis curves of MMC, Captex and CAP-MMC (Figure S2)

Band position and assignments (vibration and molecule) for the ATR FTIR spectra (Table S3)

Fourier-transform infrared spectroscopy spectra of during three months' storage (Figure S3)

Storage stability of degassed MMC (Table S4)

Evaluation of loading efficiency of LBF-MMC for different loading techniques (Table S5)

Scanning electron microscopy images LBF-MMC (Figure S4)

Calculation of lipid content in CAP-MMC

On the basis of the weight loss determined in the thermal gravimetric analysis (TGA), the following equations were used to calculate the Captex content in the CAP-MMC:

$$\%\ \mathrm{MMC} = \frac{\%\ \mathrm{MMC}_{200^{o}\mathrm{C}}}{\%\ \mathrm{MMC}_{800^{o}\mathrm{C}}} * \%\mathrm{CaptexMMC}_{200^{o}\mathrm{C}} \quad \text{(S1)}$$

$$\%\ \mathrm{Captex} = \frac{\mathrm{CaptexMMC}_{200^{o}\mathrm{C}} - \%\ \mathrm{MMC}}{\mathrm{CaptexMMC}_{200^{o}\mathrm{C}}} \quad \text{(S2)}$$

%MMC200/800˚C: Recorded weight percentage of MMC at 200/800˚C
%CaptexMMC200˚C: Recorded weight percentage of Captex-MMC at 200˚C

Table S1. Assignments of chemical shift of the ^{1}H NMR signal of the main protons of glycerides and free fatty acids during the in vitro digestion of Captex and CAP-MMC. More details can be found in previous work by Joyce et al. (2016) and Nieva-Echevarría et al. (2014, 2015).[1–3]

Signal	Chemical shift (ppm)	Multiplicity	Compounds
**a**	0.88-0.89	t	Acyl groups and FA
**b**	1.19-1.42	m	Acyl groups and FA
**c**	1.61-1.72	m	Acyl groups and FA
**d**	1.92-2.15	m	Acyl groups and FA
**e**	2.26-2.38	m	Acyl groups and FA
**f**	2.37-2.44	m	Acyl groups in TG
**g**	2.77-2.90	t	Acyl groups and FA
**h**	3.65	d	Glyceryl groups in 1-MG
**i**	3.73	d	Glyceryl groups in 1,2-DG
**j**	3.84	d	Glyceryl groups in 2-MG
**k**	3.94	m	Glyceryl groups in 1-MG
**l**	4.10-4.18<br>4.26-4.40	dd,dd	Glyceryl groups in TG
**m**	4.28	ddd	Glyceryl groups in 1,2-DG
**n**	5.08	m	Glyceryl groups in 1,2-DG
**o**	5.27	m	Glyceryl groups in TG
**p**	5.28-5.46	m	Acyl groups and FA

Abbreviations: d: doublet, t: triplet, m: multiplet, FA: fatty acid, TG: triglyceride, 1-MG: 1-monoglyceride, 1,2-DG: 1,2-diglyceride, 2-MG: 2-monoglyceride.

Calculations of the molar percentage of the different lipolytic molecules in the ^{1}H NMR samples

$$N_{MG} = \frac{A_{3.82-3.86}}{4} + \frac{A_{3.64-3.67}}{2} \quad \text{(S3)}$$

$$N_{DG} = \frac{A_{3.72-3.75}}{2} \quad \text{(S4)}$$

$$N_{TG} = \frac{2A_{4.26-4.32} - A_{3.72-3.75}}{4} \quad \text{(S5)}$$

$$N_{FFA} = \frac{A_{2.26-2.40} - 6N_{TG} - 4N_{DG} - 2N_{MG}}{2} \quad \text{(S6)}$$

In the stability study, the monoglycerides content was calculated using equation 8, as this peak was clear and distinguishable in these samples.

$$N_{MG} = \frac{A_{3.82-3.86}}{4} + A_{3.91-3.96} \quad \text{(S7)}$$

On the basis of the acetyl groups supported on different glyceride structures (TG, DG, and MG) and the molar percentage of free fatty acids (FFA), the molar percentage of the different lipolytic molecules was calculated using equations 9-12 (Joyce et al., 2016, and Nieva-Echevarría et al., 2014, 2015). [1–3]

$$MG\% = \frac{100N_{MG}}{N_{total}} \quad \text{(S8)}$$

$$DG\% = \frac{100(2N_{DG})}{N_{total}} \quad \text{(S9)}$$

$$TG\% = \frac{100(3N_{TG})}{N_{total}} \quad \text{(S10)}$$

$$FFA\% = \frac{100(N_{FFA})}{N_{total}} \quad \text{(S11)}$$

$$N_{total} = 3N_{TG} + 2N_{DG} + N_{MG} + N_{FFA} \quad \text{(S12)}$$

Reference samples of Captex:

Approximately 20 mg of Captex was dissolved in 800 µl deuterated chloroform, and transferred to an NMR tube. The composition of the lipid components were evaluated using ^{1}H NMR (see section 2.5). No peaks indicating impurities were observed in the spectra of the lipid.

Table S2: Molar percentage of the different lipid components in reference sample of Captex (n=3).

Lipid component	Molar percentage (%)
Triglycerides	97.06±0.08
Diglycerides	0.57±0.01
Free fatty acids	2.38±0.09

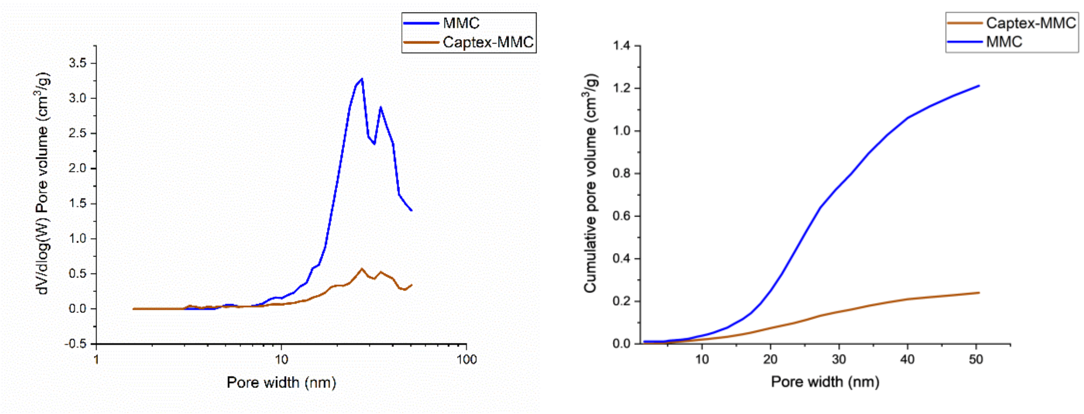

(**a**) (**b**)

Figure S1. Nitrogen sorption analysis of MMC (blue line) and CAP-MMC (brown line). (a) Pore size distribution (V=pore volume; W=pore width). (b) Cumulative pore volume.

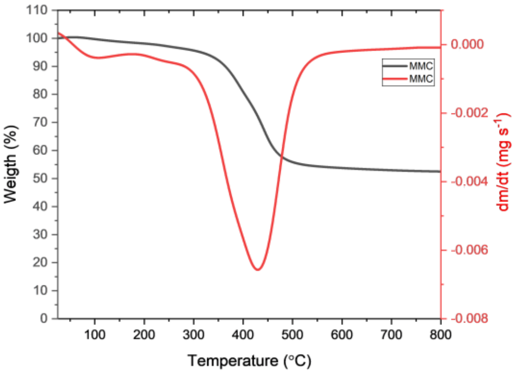

(**a**)

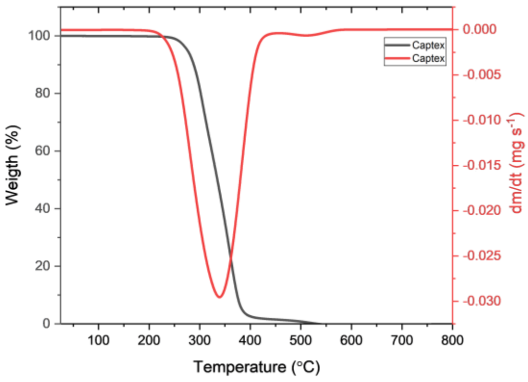

(**b**)

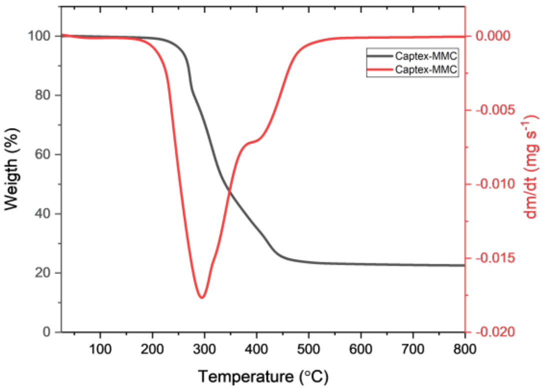

(**c**)

Figure S2. Normalized mass thermal gravimetric analysis curves of (**a**) MMC, (**b**) Captex, and (**c**) CAP-MMC. The x-axis show the changes in mass (%), and the y-axis displays the derivative of the changes in mass over time (mg/s). The CAP-MMC starts decomposing at around 200° C, and displays a two-phasic pattern in which first the lipid starts decomposing, then the MMC. This is also seen from the decomposition rate (dm/dt), where the initial peak correlates with that of the lipid, and the shoulder on the peak is consistent with that of the MMC.

Table S3. Band position and assignments (vibration and molecule) for the ATR FTIR spectra acquired for MMC, Captex and CAP-MMC.

**Band**	**Vibration**	**Reference**	**Position/cm^{-1}**		
			MMC	Captex	CAP-MMC
**a**	-CH2	[4]		724	722
**b**	-C-O	[5,6]	854		853
**c**	-C-O	[4]		1154	1155
**d**	-C-O	[5,6]	1431		1430
**e**	-CH2	[4]		1461	
**f**	-C=O ester	[4]		1740	1742
**g**	$-CH_3$ and $-CH_{2\ sym}$	[4]		2854	2855
	$-CH_3$ and $-CH_{2\ asym}$			2923/2954	2924/2955
**h**	-OH groups (hydrogen-bonded)	[5,6]	2500-3700		2500-3700

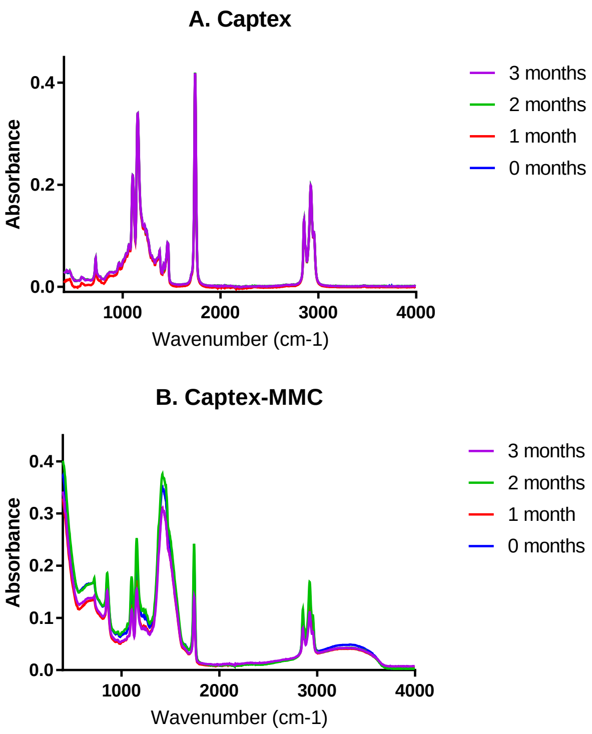

Figure S3. Fourier-transform infrared spectroscopy spectra of (a) Captex, and (b) CAP-MMC, during three months' storage in dry conditions.

Storage stability of degassed MMC

Degassed MMC was loaded with Captex in a 1:1 w/w ratio via physical adsorption. The sample was stored in a dessicator containing phosphorus pentoxide and silica gel (<5% RH/25 °C) at room temperature during one month. Samples were prepared and analyzed using ^{1}H NMR as described in section 2.6 in the main text. An FTIR spectrum was also collected of the CAP-MMC, which was consistent with spectra from non-degassed MMC.

Table S4: Molar percentage of the different lipid components of degassed MMC immediately after loading with Captex and after one month's storage (n=3).

	Molar percentage (%)	
**Lipid component**	0 months	1 month
Triglycerides	91.52±2.08	81.13±0.84
**Diglyderieds**	1.34±0.24	2.79±0.21
Monoglycerides	1.01±1.03	4.94±1.09
Free fatty acids	6.13±1.75	11.15±2.06

TableS5: Evaluation of loading efficiency for different loading techniques. The LBF was added to the MMC in the below-stated weight/weight ratio (*w/w*). The highest loading degree (green) was obtained when freeze drying was used to produce the lipid-loaded carrier.

MMC:LBF (w/w)	Physical adsorption	Solvent immersion	Freeze drying
2:1	+	+	+
1:1	+	+	+
1:1.5	+	+	+
1:2	-	-	+

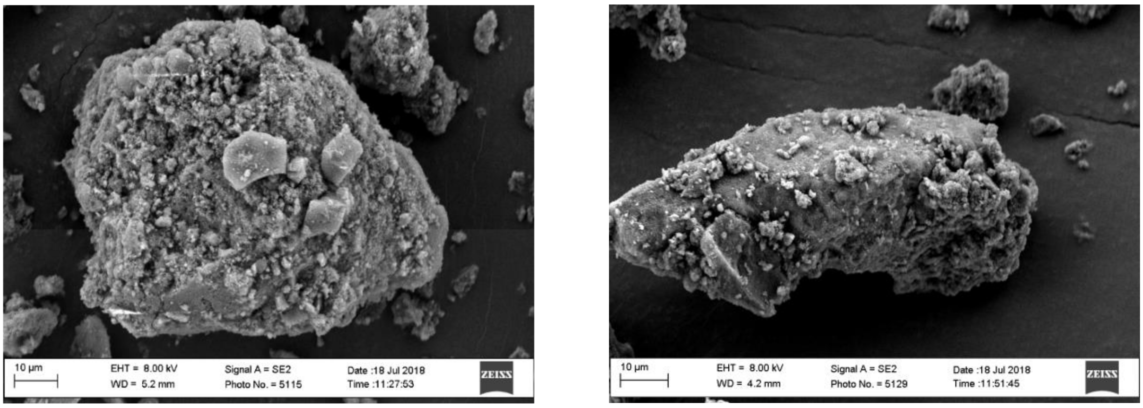

(**a**) (**b**)

Figure S4. Scanning electron microscopy images of (**a**) MMC and (**b**) LBF-loaded MMC, manufactured via freeze drying (1:2 w/w ratio MMC:LBF). No morphological changes were observed on the surface of the MMC after loading with the lipid.

References

1. Joyce, P.; J. Barnes, T.; J. Boyd, B.; A. Prestidge, C. Porous nanostructure controls kinetics, disposition and self-assembly structure of lipid digestion products. *RSC Adv.* **2016**, *6*, 78385–78395.
2. Nieva-Echevarría, B.; Goicoechea, E.; Manzanos, M.J.; Guillén, M.D. Usefulness of 1H NMR in assessing the extent of lipid digestion. *Food Chem.* **2015**, *179*, 182–190.
3. Nieva-Echevarría, B.; Goicoechea, E.; Manzanos, M.J.; Guillén, M.D. A method based on 1H NMR spectral data useful to evaluate the hydrolysis level in complex lipid mixtures. *Food Res. Int.* **2014**, *66*, 379–387.
4. Laurens, L.M.L.; Wolfrum, E.J. Feasibility of Spectroscopic Characterization of Algal Lipids: Chemometric Correlation of NIR and FTIR Spectra with Exogenous Lipids in Algal Biomass. *Bioenerg. Res.* **2011**, *4*, 22–35.
5. Cheung, O.; Zhang, P.; Frykstrand, S.; Zheng, H.; Yang, T.; Sommariva, M.; Zou, X.; Strømme, M. Nanostructure and pore size control of template-free synthesised mesoporous magnesium carbonate. *RSC Adv.* **2016**, *6*, 74241–74249.
6. Frykstrand, S.; Forsgren, J.; Mihranyan, A.; Strømme, M. On the pore forming mechanism of Upsalite, a micro- and mesoporous magnesium carbonate. *Microporous Mesoporous Mater.* **2014**, *190*, 99–104.
